# CPSF3 inhibition blocks pancreatic cancer cell proliferation through disruption of core histone mRNA processing

**DOI:** 10.1261/rna.079931.123

**Published:** 2024-03

**Authors:** Abdulrahman A. Alahmari, Aditi H. Chaubey, Venkata S. Jonnakuti, Arwen A. Tisdale, Carla D. Schwarz, Abigail C. Cornwell, Kathryn E. Maraszek, Emily J. Paterson, Minsuh Kim, Swati Venkat, Eduardo Cortes Gomez, Jianmin Wang, Katerina V. Gurova, Hari Krishna Yalamanchili, Michael E. Feigin

**Affiliations:** 1 Department of Pharmacology and Therapeutics, Roswell Park Comprehensive Cancer Center, Buffalo, New York 14203, USA; 2 Department of Medical Laboratory Sciences, College of Applied Medical Sciences, Prince Sattam Bin Abdulaziz University, Alkharj 11942, Saudi Arabia; 3 Department of Pediatrics, Baylor College of Medicine, Houston, Texas 77030, USA; 4 Program in Quantitative and Computational Biology, Baylor College of Medicine, Houston, Texas 77030, USA; 5 Medical Scientist Training Program, Baylor College of Medicine, Houston, Texas 77030, USA; 6 Department of Biostatistics and Bioinformatics, Roswell Park Comprehensive Cancer Center, Buffalo, New York 14203, USA; 7 Department of Cell Stress Biology, Roswell Park Comprehensive Cancer Center, Buffalo, New York 14203, USA; 8 Jan and Dan Duncan Neurological Research Institute at Texas Children's Hospital, Houston, Texas 77030, USA; 9 USDA/ARS Children's Nutrition Research Center, Department of Pediatrics, Baylor College of Medicine, Houston, Texas 77030, USA

**Keywords:** CPSF3, JTE-607, alternative polyadenylation, histone processing, chromatin stability

## Abstract

Pancreatic ductal adenocarcinoma (PDAC) is a lethal disease with limited effective treatment options, potentiating the importance of uncovering novel drug targets. Here, we target cleavage and polyadenylation specificity factor 3 (CPSF3), the 3′ endonuclease that catalyzes mRNA cleavage during polyadenylation and histone mRNA processing. We find that *CPSF3* is highly expressed in PDAC and is associated with poor prognosis. *CPSF3* knockdown blocks PDAC cell proliferation and colony formation in vitro and tumor growth in vivo. Chemical inhibition of CPSF3 by the small molecule JTE-607 also attenuates PDAC cell proliferation and colony formation, while it has no effect on cell proliferation of nontransformed immortalized control pancreatic cells. Mechanistically, JTE-607 induces transcriptional readthrough in replication-dependent histones, reduces core histone expression, destabilizes chromatin structure, and arrests cells in the S-phase of the cell cycle. Therefore, CPSF3 represents a potential therapeutic target for the treatment of PDAC.

## INTRODUCTION

Pancreatic ductal adenocarcinoma (PDAC) is the third leading cause of cancer-related deaths with a 5-yr survival rate of 12%, due in part to the lack of effective treatment options (Siegel et al. 2023). PDAC is primarily driven by mutations in the oncogene *KRAS* and several tumor suppressors, including *TP53*, *CDKN2A*, and *SMAD4* (Kleeff et al. 2016). However, as clinically effective modulators of the activity of these proteins are not currently available, identification of novel targets amenable to small molecule inhibition is a critical undertaking. Recently, large-scale RNA-sequencing efforts of PDAC tumors have revealed widespread dysregulation of oncogenic gene expression, allowing the characterization of several PDAC subtypes and phenotypic states (Collisson et al. 2011; Moffitt et al. 2015; Bailey et al. 2016; Peng et al. 2019). These gene expression changes are critical for driving tumor phenotypes, including metastatic progression (Shankar et al. 2016; Roe et al. 2017; Abel et al. 2018; Wang et al. 2019; Sodir et al. 2020). While these gene expression changes have been extensively cataloged, the mechanisms underlying this transcriptional heterogeneity remain largely unknown (Venkat et al. 2021). We propose that targeting these drivers of dysregulated gene expression represents an opportunity to reverse widespread oncogenic activity in transformed cells.

One such gene regulatory process that has been implicated in cancer is mRNA processing, a step that is crucial for the maturity of newly transcribed RNAs. For most human genes, nascent RNAs undergo cleavage and polyadenylation, or CPA. Because most genes have multiple polyadenylation recognition sites (PASs) within the 3′ untranslated region (UTR), the choice of where mRNA is cleaved and polyadenylated can generate distinct transcript isoforms with different 3′-UTR lengths, ultimately affecting mRNA stability, localization, and translation (Gruber and Zavolan 2019). This process is called alternative polyadenylation, or APA, and is widely dysregulated in cancer (Masamha and Wagner 2018; Gruber and Zavolan 2019; Yuan et al. 2021). Recently, we identified widespread APA alterations in PDAC patients that are associated with functional changes in both gene and protein expression of growth-promoting genes (Venkat et al. 2020). Unlike polyadenylated genes, a class of histone genes is processed on the mRNA level by cleavage but not polyadenylation. These histones are replication-dependent (RD) and are crucial for cell proliferation. While CPA and histone mRNA processing are regulated by two different complexes, some proteins are in fact important regulators of both processes. One such protein that is the focus of our study is cleavage and polyadenylation specificity factor 3 (CPSF3) (Sullivan et al. 2009b), the endonuclease responsible for the cleavage of mRNAs. As a part of the CPA complex, CPSF3 cooperates with other CPA factors to cleave the mRNA before the addition of the poly(A) tail. As part of the histone cleavage complex (HCC), however, CPSF3 cleaves pre-mRNAs of RD core histones, but these pre-mRNAs do not get polyadenylated. Both CPA and histone mRNA processing are important biological processes for cell proliferation and survival. The fact that CPSF3 is an enzyme opens the possibility of its pharmacological targeting. Recently, CPSF3 was identified as the target of the small molecule JTE-607 (Kakegawa et al. 2019; Ross et al. 2020). JTE-607 is hydrolyzed into an active compound that directly interacts with the CPSF3 interfacial cavity (Ross et al. 2020). This interaction inhibits CPSF3 catalytic activity leading to accumulation of unprocessed newly synthesized pre-mRNAs. JTE-607 induces apoptosis of human acute myeloid leukemia (AML) and Ewing's sarcoma cells in vitro and prolongs survival of tumor-bearing mice in xenograft models in vivo (Uesato et al. 2006; Tajima et al. 2010). JTE-607 inhibits migration, invasion, and self-renewal of breast cancer cells (Liu et al. 2022). Notably, administration of JTE-607 in healthy volunteers demonstrated the safety of this compound in humans, with no severe adverse events reported (Borozdenkova et al. 2011). However, the role of CPSF3 and the effect of JTE-607 in epithelial cancers remain largely unknown.

Here, we show that knockdown and/or inhibition of CPSF3 attenuates PDAC cell proliferation in vitro and in vivo. We find that *CPSF3* is highly expressed in PDAC patients and is a predictor of poor outcome. We demonstrate that small molecule inhibition of CPSF3 by JTE-607 selectively attenuates the proliferation of PDAC cells but not immortalized control cells. Additionally, we conduct a global analysis of CPSF3 disruption in PDAC, uncovering gene regulatory mechanisms that distinctly affect PDAC cells upon either *CPSF3* knockdown or inhibition. We uncover that JTE-607 dysregulates RD histones, destabilizes chromatin structure, and arrests cells in the S-phase of the cell cycle. Overall, our findings uncover new functions of CPSF3 in cancer and nominate CPSF3 as a novel therapeutic target in PDAC.

## RESULTS

### *CPSF3* is up-regulated in human PDAC and required for PDAC cell proliferation

**FIGURE 1.**
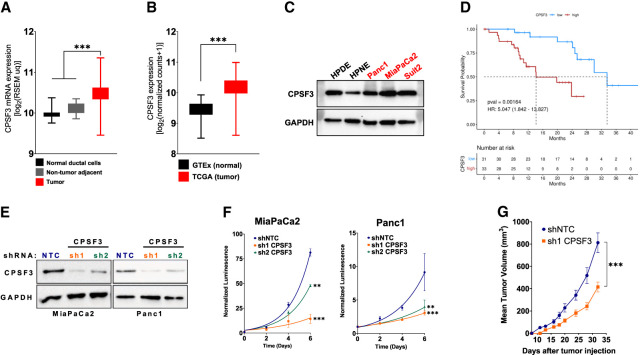
*CPSF3* is highly expressed in PDAC and is required for PDAC cell proliferation. (*A*) *CPSF3* mRNA expression from CPTAC PDAC patient data. Whiskers indicate minimum and maximum data points. (***) *P* < 0.0001, ordinary one-way ANOVA with Tukey multiple comparisons test. (*B*) *CPSF3* mRNA expression from PDAC patient data (TCGA) as compared to normal pancreas (GTEx). Whiskers indicate minimum and maximum data points. (***) *P* < 0.0001, unpaired *t*-test with Welch's correction. (*C*) Immunoblot of CPSF3 in immortalized control pancreatic epithelial cells (black) and PDAC cells (red). (*D*) Kaplan–Meier survival curves of PDAC patients with high (red) and low (blue) *CPSF3* mRNA levels. Data were obtained from the CPTAC database. (*E*) Immunoblot of CPSF3 in shNTC, sh1, and sh2 CPSF3 knockdown cells. (*F*) Proliferation rates at days 0, 2, 4, and 6 of shNTC (blue), sh1 (orange), and sh2 (green) CPSF3 knockdown cells. (**) *P* < 0.01, (***) *P* < 0.001; two-way ANOVA with Dunnett's multiple comparisons test. (*G*) Mean tumor volume (mm^3^) of CPSF3 knockdown (orange) and control (blue) MiaPaCa2 tumors. (***) *P* < 0.001, two-way ANOVA.

To determine the clinical significance of *CPSF3* expression in PDAC, we first analyzed gene expression data from the Clinical Proteomic Tumor Analysis Consortium (CPTAC) (Cao et al. 2021). *CPSF3* expression was significantly higher in PDAC tumors (*n* = 135), as compared with non-tumor adjacent tissues (*n* = 18) and normal pancreata (*n* = 7) (Fig. 1A). Consistent with this finding, *CPSF3* expression was also significantly higher in the pancreatic adenocarcinoma (PAAD) data set from The Cancer Genome Atlas (TCGA) (*n* = 147) as compared to normal pancreata (*n* = 165) from the Genotype-Tissue Expression (GTEx) project (Fig. 1B). We then sought to assess *CPSF3* expression status in our cell line models. In agreement with the clinical data, we found that *CPSF3* is up-regulated in PDAC cell lines (MiaPaCa2, Suit2, Panc1) as compared to nontransformed immortalized pancreatic epithelial cells (HPNE and HPDE; from now on referred to as immortalized control cells) by western blot (WB) and RT-qPCR (Fig. 1C; Supplemental Fig. S1A). Other CPA factors were also up-regulated in our PDAC cell lines compared to immortalized control HPNE cells (Supplemental Fig. S1B–I). This is consistent with our previous report where multiple CPA factors are up-regulated in PDAC patients (Venkat et al. 2020). We chose to focus on CPSF3 as it is an enzyme and therefore is a potential druggable target. We then sought to assess the relationship between *CPSF3* expression and PDAC patient outcome. Patients with high *CPSF3* expression had significantly worse overall survival than patients with low *CPSF3* expression (*P* = 0.00164, hazard ratio 5.047 [1.842–13.827]). Specifically, patients in the top quartile of *CPSF3* expression had a median survival of 14.2 mo, while those in the bottom quartile of *CPSF3* expression had a median survival of 33.5 mo (Fig. 1D). Therefore, *CPSF3* is highly expressed in PDAC, high expression correlates with poor patient outcome, and our cell models are appropriate for mechanistic studies.

To define the functional role of *CPSF3* in PDAC, we first took a genetic approach and generated stable *CPSF3* knockdown MiaPaCa2 and Panc1 cells. We used two different short-hairpin RNAs (sh1 and sh2) targeting *CPSF3*, and a nontargeting control (shNTC). Successful knockdown of *CPSF3* was confirmed at the protein and RNA level by WB and RT-qPCR, respectively, with sh1 cells having the highest level of knockdown in both cell lines (Fig. 1E; Supplemental Fig. S2A). We then examined the effect of *CPSF3* knockdown on cell proliferation and colony formation capability. *CPSF3* knockdown significantly attenuated proliferation as compared with shNTC controls in both MiaPaCa2 and Panc1 cells (Fig. 1F). *CPSF3* knockdown also significantly decreased colony formation (Supplemental Fig. S2B,C). In both the proliferation and colony formation assays, and in both PDAC cell lines, sh1 CPSF3 had the strongest phenotype, consistent with higher levels of *CPSF3* knockdown. In contrast, knockdown of *CPSF3* in immortalized HPNE cells had no effect on proliferation (Supplemental Fig. S2D,E). Next, we sought to determine the requirement for CPSF3 in PDAC tumor growth in vivo. We implanted MiaPaCa2 cells (either shNTC or sh1 CPSF3, 5 × 10^5^ per mouse) subcutaneously into the flanks of NOD/SCID/IL2Rγ^−/−^ (NSG) mice. *CPSF3* knockdown tumors grew significantly slower, and weighed significantly less at the end point, than shNTC tumors (Fig. 1G; Supplemental Fig. S3A,B). No changes in tumor histopathology were noted by hematoxylin and eosin (H&E) staining (Supplemental Fig. S3C). Immunohistochemical (IHC) analysis revealed that CPSF3 knockdown was maintained in vivo (Supplemental Fig. S3D). Finally, IHC for Ki67 revealed a significant decrease in proliferation in *CPSF3* knockdown tumors as compared with shNTC controls (Supplemental Fig. S3E). Overall, these data support the requirement for CPSF3 in PDAC cell proliferation and tumor growth.

### PDAC cells are sensitive to the chemical inhibition of CPSF3

**FIGURE 2.**
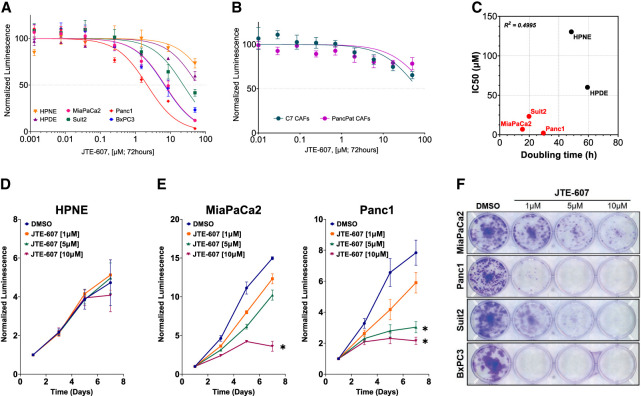
PDAC cell lines are sensitive to CPSF3 inhibition by JTE-607. (*A*) IC50 of JTE-607 on immortalized control (HPNE and HPDE) and PDAC (MiaPaCa2, Panc1, Suit2, BxPC3) cell lines after 72 h of treatment. (*B*) IC50 of JTE-607 on human fibroblast C7 and PancPat CAFs after 72 h of treatment. (*C*) Association between doubling time and IC50 of JTE-607 in pancreatic cell lines. Red denotes PDAC cells while black denotes immortalized control cell lines. *R*^2^ = 0.4995. (*D, E*) Proliferation rates at days 0, 2, 4, and 6 of immortalized control and PDAC cell lines after treatment with escalating concentrations of JTE-607. (*) *P* < 0.05; two-way ANOVA with Dunnett's multiple comparisons test. Data are shown as mean ± SEM. (*F*) Clonogenic growth assay of PDAC cell lines after treatment with increasing concentration of JTE-607.

CPSF3 was recently identified as the target for the small molecule JTE-607. JTE-607 is a prodrug that, when metabolized by the ester hydrolyzing enzyme carboxylesterase 1 (CES1), binds to CPSF3 and inhibits its catalytic activity, impairing the processing of newly synthesized mRNAs (Ross et al. 2020). As genetic depletion of *CPSF3* attenuated PDAC cell proliferation (Fig. 1), we hypothesized that pharmacologic inhibition of CPSF3 with JTE-607 could represent a novel therapeutic approach in PDAC. We therefore examined the sensitivity of multiple human pancreatic cell lines, both immortalized control cells and PDAC, to JTE-607 in a 72-h dose–response cell viability assay. Immortalized control pancreatic epithelial cells (HPNE, IC50 = 130.4 μM; HPDE, IC50 = 60.11 μM) and human cancer-associated fibroblast cell lines (C7 CAF, IC50 = 70.04 μM; PancPat CAFs, IC50 = 114.2 μM) were not sensitive to JTE-607 (Fig. 2A,B). In contrast, human PDAC cell lines displayed a range of sensitivities to JTE-607, with Panc1 cells being the most sensitive (IC50 = 2.163 μM) (Fig. 2A). Importantly, the relationship between cell line doubling time and JTE-607 sensitivity shows that sensitivity to JTE-607 was associated with proliferation rate (Fig. 2C). Next, we determined the effect of JTE-607 on cell proliferation by treating cells with increasing concentrations of JTE-607 and assessing cell viability in a time-dependent fashion (Fig. 2D,E). JTE-607 had no effect on proliferation in HPNE cells (Fig. 2D). However, the proliferation of MiaPaCa2 and Panc1 PDAC cells was significantly attenuated by JTE-607, in a dose-dependent manner (Fig. 2E). Finally, we tested the effect of JTE-607 on colony formation in PDAC cell lines. JTE-607 significantly decreased colony formation in all PDAC cell lines tested (Fig. 2F; Supplemental Fig. S4A–D). Therefore, JTE-607 selectively attenuates the proliferation of PDAC cells over immortalized control pancreatic cells.

### mRNA 3′-end processing is distinct between knockdown and chemical inhibition of CPSF3

Because JTE-607 inhibits CPSF3 catalytic activity without inducing target degradation, we sought to understand if the function of CPSF3 is distinct between knockdown and inhibition. As CPSF3 is an integral component of the CPA complex and the HCC (Wagner et al. 2007; Sullivan et al. 2009b; Yang et al. 2020), we hypothesized that CPSF3 disruption would affect both APA and histone mRNA processing. To test this hypothesis, we subjected *CPSF3* knockdown and JTE-607-treated Panc1 cells to RNA-sequencing (whole transcriptome sequencing with ribosomal RNA depletion and primed with random priming). Next, we performed APA analysis using polyAMiner-Bulk to uncover significantly altered changes in 3′-UTR length (Yalamanchili et al. 2020; Jonnakuti et al. 2023). Briefly, polyAMiner-Bulk detects APA alterations from bulk RNA-seq data (see Materials and Methods for details) by generating a poly(A) index score (PolyAIndex) for each gene based on the relative abundances of 3′-UTR long and short forms. Cleavage at a proximal polyadenylation signal (pPAS) generates a short 3′ UTR, while cleavage at a distal polyadenylation signal (dPAS) generates a long 3′ UTR. A negative PolyAIndex indicates a shortening event, and a positive PolyAIndex indicates 3′-UTR lengthening. To identify differential APA genes (DAGs) with minimum false positives/negatives and better understand the differences between knockdown and inhibition, we chose a stringent PolyAIndex threshold (−0.5 > PolyAIndex > 0.5; *P*adj < 0.05) (Supplemental Table S1). In the *CPSF3* knockdown cells, PolyAMiner-Bulk detected 85 significant DAGs, of which 43 genes underwent 3′-UTR lengthening (PolyAIndex > 0.5; *P*adj < 0.05) and 42 genes underwent 3′-UTR shortening (PolyAIndex < −0.5; *P*adj < 0.05) (Supplemental Fig. S5A). In the CPSF3 inhibition model, PolyAMiner-Bulk detected 174 significant DAGs, of which 138 underwent 3′-UTR lengthening (PolyAIndex > 0.5; *P*adj < 0.05) and 36 genes underwent 3′-UTR shortening (PolyAIndex < −0.5; *P*adj < 0.05) (Supplemental Fig. S5B). Of note, JTE-607 treatment exhibited more DAGs than *CPSF3* knockdown, with genes undergoing lengthening events being the most predominant. Surprisingly, however, the DAGs identified in both *CPSF3* knockdown and inhibition do not converge, with only two shared DAGs altered in the same direction between both conditions (Supplemental Fig. S5C).

To determine if these distinct patterns are due to differences in CPA complex stability upon *CPSF3* knockdown or inhibition, we performed immunoprecipitation (IP) experiments to pull down multiple CPA complexes. The CPA machinery is composed of multiple complexes including the CPSF complex, the cleavage stimulation factor (CSTF) complex, and the cleavage factor (CFI and CFII) complexes. The CPSF complex forms two subcomplexes, the mammalian polyadenylation specificity factor (mPSF) containing CPSF1, WDR33, FIP1, and CPSF4, which recognizes the AAUAAA PAS, and the mammalian cleavage factor (mCF) subcomplex containing CPSF2, CPSF3, and Symplekin, which possesses endonucleolytic activity (Shi and Manley 2015). We found that *CPSF3* knockdown, but not inhibition, destabilizes the CPA complex (Supplemental Fig. S6A–C). The amount of CPSF2 and CPSF3 bound to CPSF4 decreases upon *CPSF3* knockdown, consistent with their heterodimer function (Supplemental Fig. S6A). The other CPA factors probed show increased basal protein levels upon *CPSF3* knockdown (Supplemental Fig. S6A, input columns). Protein levels of CSTF2 and NUDT21, which bind to U/GU-rich elements downstream from PAS and UGUA-rich elements upstream of PAS, respectively, both increase upon *CPSF3* knockdown. Therefore, the stability of the CPA complex upon *CPSF3* knockdown may at least partially be attributed to dysregulated basal protein levels of multiple CPA factors. On the other hand, CPSF3 inhibition did not affect the stability or basal protein levels of CPA complexes (Supplemental Fig. S6B). Of note, knockdown or inhibition of CPSF3 did not largely affect CPA factor expression on the mRNA level (Supplemental Fig. S6D,E), indicating that the effect of *CPSF3* knockdown on CPA factor expression is not transcriptional.

To better understand the difference between knockdown and inhibition, we next asked which type of *cis*-elements are regulated in both conditions, thus influencing PAS selection. Multiple *cis*-elements have been shown to promote APA in an opposing manner. For example, the CPA factor FIP1 binds to an A-rich sequence upstream of the canonical AAUAAA PAS (upstream sequence element, or USE) and promotes the usage of proximal PASs, thus inducing the shortening of genes (Lackford et al. 2014). In contrast, NUDT21, the small subunit of cleavage factor 1, binds to UGUA-containing USE. When binding to UGUA-containing USE near distal PASs, NUDT21 prevents the CPSF subunits from interacting with proximal PASs, thus inducing lengthening of genes (Brown and Gilmartin 2003; Martin et al. 2012). To address the 3′-end processing differences between knockdown and inhibition, we performed two independent motif enrichment analyses. First, we examined the distribution of the UGUA motif within the 3′ UTR of genes that underwent shortening in both conditions. We found significant enrichment for UGUA motifs near distal PASs (∼25–50 bp upstream) compared to the proximal PASs within the 3′ UTR of genes that exhibit shortening changes following *CPSF3* knockdown (Supplemental Fig. S7A, pink highlight). These results indicate that CPSF3 strongly binds at distal PASs of the unique 3′-UTR shortened genes and that *CPSF3* knockdown shifts this PAS selection to a proximal PAS. On the other hand, CPSF3 inhibition by JTE-607 did not show consistent distribution patterns of the UGUA motif (Supplemental Fig. S7B), suggesting that enzymatic inhibition of CPSF3 may rely on other *cis*-elements to direct PAS selection. To identify which *cis*-elements are enriched upon both *CPSF3* knockdown and inhibition in an unbiased manner, we selected the genes that are uniquely identified as undergoing 3′-UTR lengthening or shortening in both experiments and performed motif enrichment analysis within the 100 bp upstream and downstream (50 bp in each direction) of the most proximal and most distal PASs (refer to Materials and Methods in the Supplemental file for more details). We found distinct motif enrichment across *CPSF3* knockdown and inhibition at both proximal and distal PASs (Supplemental Fig. S7C,D). For example, genes undergoing shortening upon *CPSF3* knockdown were enriched for the canonical PAS AATAAA within the pPAS (Supplemental Fig. S7C, pink highlight). In contrast, a similar AATAAA sequence was enriched within the pPAS of lengthened genes upon JTE-607 treatment (Supplemental Fig. S7D, blue highlight). The fact that *CPSF3* knockdown and inhibition DAGs show the consensus AATAAA signal in distinct sets (lengthened and shortened, respectively) suggests diverse polyadenylation site selection. This is also substantiated by the poor overlap of *CPSF3* knockdown and inhibition DAGs shown in Supplemental Figure S5C. Therefore, this difference suggests selection for different PASs, thus supporting the notion that *CPSF3* knockdown and inhibition differentially affect the site of polyadenylation.

### JTE-607 inhibits expression of replication-dependent histones

**FIGURE 3.**
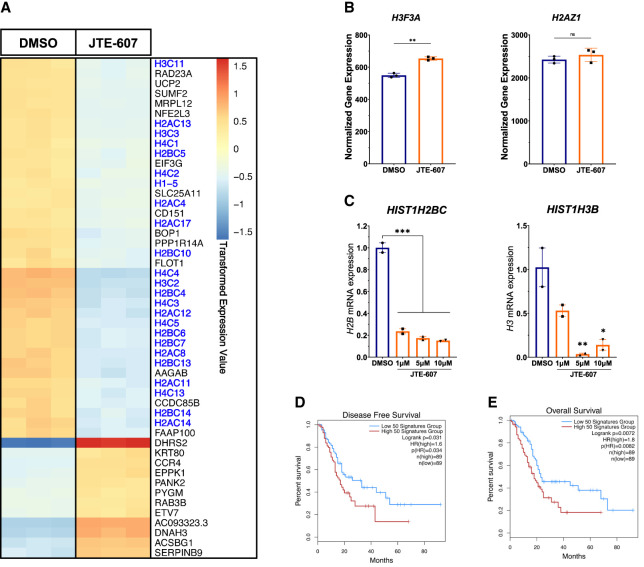
JTE-607 decreases gene expression of RD histones. (*A*) Heatmap of top differentially expressed genes after 24 h of 10 µM JTE-607 treatment. RD histones are colored in blue. Expression is plotted as transformed expression value. (*B*) DSeq2 normalized counts of *H3F3A* and *H2AZ1* histone variants (RI) in Panc1 cells treated with 10 µM JTE-607 for 24 h. (**) *P* < 0.001. (*C*) mRNA expression of *H2B* (*HIST1H2BC*) and *H3* (*HIST1H3B*) in MiaPaCa2 cells treated with JTE-607. (*) *P* < 0.05, (**) *P* < 0.01, (***) *P* < 0.001, ordinary one-way ANOVA with Dunnett's multiple comparisons test. (*D*, *E*) Survival analyses of low (blue) and high (red) expression of the RD histone signature (50 genes) in the TCGA-PAAD data set. Signature genes were uploaded to GEPIA2 to assess disease-free (*D*) and overall survival (*E*) based on median.

We next sought to understand the mechanism by which CPSF3 disruption attenuates PDAC cell proliferation. Recently, we reported widespread APA shortening events in PDAC patients that are associated with oncogenic functions (Venkat et al. 2020). Therefore, we asked whether CPSF3 disruption would reverse the APA patterns of those growth-promoting genes. However, neither *CPSF3* knockdown nor inhibition altered the APA patterns of these genes (Supplemental Fig. S8A). In fact, few genes were altered on both the APA and gene expression levels by either *CPSF3* knockdown or inhibition (Supplemental Fig. S8B). These data suggest that PDAC phenotype is mediated by other mechanisms in our cell line models. In addition to CPA, CPSF3 controls histone mRNA processing as part of the HCC. Therefore, we sought to understand whether CPSF3 disruption affects histone processing in PDAC cells. We performed differential gene expression analysis and were intrigued to find that numerous histone genes were significantly down-regulated upon JTE-607 treatment (Fig. 3A, blue-labeled genes). Gene set enrichment analysis (GSEA) also demonstrated a dysregulation in many histone-related pathways, including histone methylation, acetylation, and deacetylation (Supplemental Fig. S9A). However, *CPSF3* knockdown did not affect histone gene expression in our cell line model (Supplemental Fig. S9B). In fact, the discrepancies between *CPSF3* knockdown and inhibition extend to the overall differential gene expression with only 119 genes being differentially expressed in both conditions (Supplemental Fig. S9C).

Histone genes are classified into two classes: replication-independent (RI) and RD histones. RI histones are processed on their mRNA 3′ end by CPA and therefore polyadenylated. In contrast, RD histone mRNAs are processed by the HCC and are not polyadenylated (Marzluff et al. 2008). RD histones are actively transcribed during DNA replication and are important for the proliferation of dividing cells. The majority of the differentially expressed histones upon CPSF3 inhibition with JTE-607 were RD histones. In contrast, RI histones were not down-regulated by JTE-607 (Fig. 3B). To validate the JTE-607-induced decrease in RD histones in another PDAC cell line, we assessed mRNA levels of two RD histones (*HIST1H2BC* and *HIST1H3B*) in MiaPaCa2 cells using RT-qPCR. Similar to Panc1 cells, JTE-607 reduced RD histone mRNA levels in MiaPaCa2 (Fig. 3C). Therefore, JTE-607 treatment decreases the expression of RD histones. Finally, we sought to determine if RD histone expression predicts patient outcomes. We generated a signature by selecting 50 RD histones and assessed PDAC patient survival based on gene expression. We found that high levels of RD histones are associated with worse disease progression (*P* = 0.031, hazard ratio = 1.6) and poor overall survival (*P* = 0.0072, hazard ratio = 1.8) in PDAC patients (Fig. 3D,E). Collectively, these results indicate that JTE-607 preferentially down-regulates RD histones.

### JTE-607 induces RD histone readthrough preferentially in PDAC cells

**FIGURE 4.**
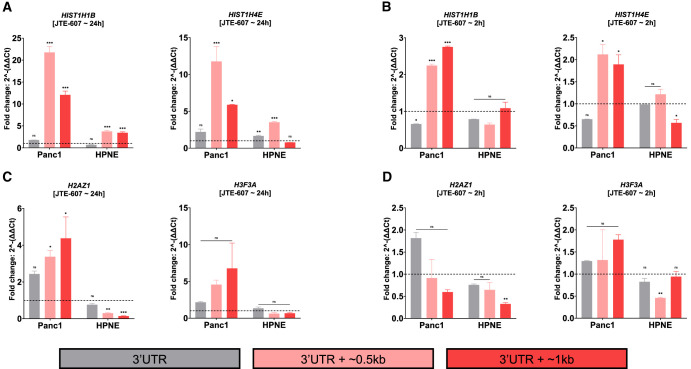
JTE-607 induces RD histone transcriptional readthrough. (*A*, *B*) Quantification of RD histone readthrough in Panc1 and HPNE cells after 24 h (*A*) and 2 h (*B*) of 10 µM JTE-607 treatment by RT-qPCR. Data were normalized to DMSO controls (dashed *horizontal* line). (*) *P* < 0.05, (**) *P* < 0.01, (***) *P* < 0.001; two-way ANOVA with Sidak's multiple comparisons test. (*C*, *D*) Quantification of RI histone readthrough in Panc1 and HPNE cells after 24 h (*C*) and 2 h (*D*) of 10 µM JTE-607 treatment by RT-qPCR. Data were normalized to DMSO controls (dashed *horizontal* line). (*) *P* < 0.05, (**) *P* < 0.01, (***) *P* < 0.001; two-way ANOVA with Sidak's multiple comparisons test.

Disruption of the HCC has been shown to induce transcriptional readthrough of histone transcripts (Wagner et al. 2007; Romeo et al. 2014). While several studies have demonstrated a role for CPSF3 in histone processing (Wagner et al. 2007; Sullivan et al. 2009b; Yang et al. 2013, 2020), the effect of chemical inhibition of CPSF3 activity on histone mRNA processing has never been biologically determined. We therefore sought to investigate whether CPSF3 inhibition induces transcriptional readthrough experimentally by RT-qPCR. We picked two RD and two RI histones that show differences beyond their 3′-end boundaries for experimental validation (Supplemental Fig. S10A,B). We then designed PCR primers to amplify different regions within and beyond the boundaries of the 3′ UTR (Supplemental Fig. S10C). We found that 24 h JTE-607 treatment significantly induced transcriptional readthrough (up to ∼20-fold change) of RD histones in Panc1 cells (Fig. 4A). However, the effect of JTE-607 on transcriptional readthrough in HPNE cells was minimal (Fig. 4A). In fact, 2 h of JTE-607 treatment were enough to induce transcriptional readthrough levels in Panc1 cells comparable to those in HPNE cells after 24 h of treatment (Fig. 4A,B). Importantly, JTE-607 did not induce transcriptional readthrough of RI histones at early or late time points in both Panc1 and HPNE cells (Fig. 4C,D). We then validated the transcriptional readthrough in another cell line model, MiaPaCa2, in a dose-dependent manner (Supplemental Fig. S10D,E). We show that JTE-607 induces significant levels of readthrough in RD histones as compared to RI histones. As *CPSF3* knockdown did not affect histone mRNA levels, we aimed to further delineate the differences between knockdown and inhibition in inducing transcriptional readthrough. We found that long-term knockdown of *CPSF3* by short-hairpin RNA (shRNA) did not induce transcriptional readthrough in both RD and RI histones (Supplemental Fig. S10F). Because stable long-term knockdown can force cells to adapt, we asked whether short-term knockdown of *CPSF3* can recapitulate the JTE-607 effect on transcriptional readthrough. We transiently silenced *CPSF3* using small-interfering RNA (siRNA) (Supplemental Fig. S10G) and found that *CPSF3* silencing did not induce transcriptional readthrough in both RD and RI histones (Supplemental Fig. S10H). Improperly processed histone mRNAs fail to be exported into the cytoplasm for translation, leading to decreased protein levels (Sullivan et al. 2009a,b; Romeo et al. 2014). Therefore, we examined RD histone protein levels upon JTE-607 treatment and found that JTE-607 reduced both H3 and H2B protein levels in a dose- and time-dependent fashion in Panc1 but not HPNE cells (Supplemental Fig. S10I,J). Next, we determined whether histone dysregulation might be transcriptionally mediated by dysregulation of transcription factors at the levels of APA or gene expression. We used MotifMap, an integrative genome-wide map of regulatory motif sites, to find putative transcription factors regulating the expression of RD histones (Daily et al. 2011). We found 51 transcription factors that have strong binding sites (1000 bp upstream of transcription start site; FDR < 0.05) within RD histone promoters (Supplemental Table S2). However, these histone transcription factors are neither APA altered nor differentially expressed upon JTE-607 treatment (Supplemental Fig. S10K,L). Taken together, these findings indicate that JTE-607 decreases RD histone expression by promoting transcriptional readthrough.

### JTE-607 destabilizes chromatin and blocks cell cycle progression

**FIGURE 5.**
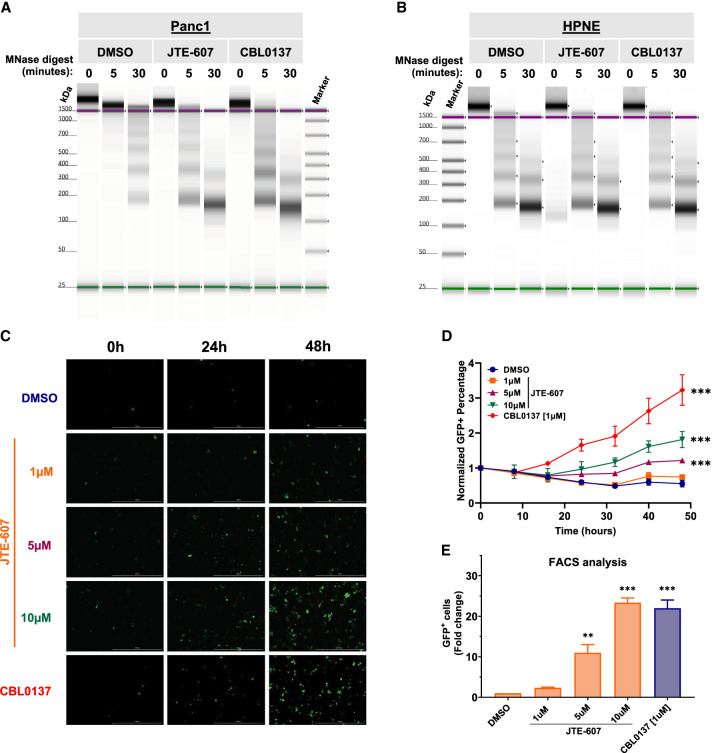
JTE-607 induces chromatin instability selectively in PDAC cells. (*A*) MNase assay of Panc1 cells treated with 10 µM JTE-607 or 1 µM CBL0137. (*B*) MNase assay of immortalized HPNE control cells treated with the CPSF3 inhibitor JTE-607 (10 µM) or CBL0137 (1 µM). (*C*) GFP + HeLa-TI cells following 10 µM JTE-607 or 1 µM CBL0137 treatment. (*D*) Fold change of GFP + HeLa-TI from *C*. (***) *P* < 0.0001; two-way ANOVA with Tukey's multiple comparisons test. (*E*) Flow cytometry analysis of GFP + HeLa-TI cells following 10 µM JTE-607 or 1 µM CBL0137 treatment. Fold change is shown as mean ± SEM of two independent experiments. (**) *P* < 0.01, (***) *P* < 0.0001, ordinary one-way ANOVA with Tukey's multiple comparisons test.

As RD histones are required for nucleosome assembly (Gunjan et al. 2005; Groth et al. 2007; Marzluff et al. 2008; Günesdogan et al. 2014), we hypothesized that JTE-607 would dysregulate chromatin dynamics. Gene ontology analysis of down-regulated genes upon JTE-607 treatment showed an enrichment for chromatin-related processes including chromatin assembly, nucleosome assembly, and nucleosome organization (Supplemental Fig. S11A). Therefore, we performed a micrococcal nuclease (MNase) assay to assess relative chromatin condensation. Using chromatin DNA, MNase digests open DNA regions that are not stably bound by proteins, thus producing nucleosome fragmentation patterns that are indicators of whether chromatin is in a condensed or relaxed state. The chromatin destabilizing agent CBL0137 was used as a positive control (Xiao et al. 2021). Panc1 cells treated with JTE-607 or CBL037 displayed rapid and complete chromatin digestion, as compared with DMSO-treated cells (Fig. 5A). After 30 min of incubation, MNase digestion released more mononucleosomes in JTE-607 (∼4 × 10^3^ normalized FU) as compared to DMSO (∼1.2 × 10^3^ normalized FU) (Supplemental Fig. S11B–E). Because HPNE cells are insensitive to JTE-607 (Fig. 2A,D), we sought to determine the impact of CPSF3 inhibition on chromatin structure in HPNE cells. In contrast to Panc1 cells, HPNE cells treated with JTE-607 or CBL037 showed no chromatin digestion as compared with DMSO-treated cells (Fig. 5B). In fact, the amount of digested mononucleosomes in HPNE cells with all treatments is comparable to DMSO-treated Panc1 cells (Supplemental Fig. S11F–I). These results suggest that JTE-607 preferentially targets cells that are in high demand for histone supplies. To assess chromatin destabilization in a living cell, we utilized the HeLa-TI cell line model that has a silenced GFP reporter within a heterochromatic region of the genome. Treatment of these cells with chromatin destabilizing agents, including CBL0137, allows derepression of GFP silencing. Therefore, we monitored GFP expression in HeLa-TI cells upon JTE-607 treatment by both fluorescence microscopy and flow cytometry. Cells treated with JTE-607 induced GFP expression to levels comparable with CBL0137 in a dose- and time-dependent manner (Fig. 5C–E).

**FIGURE 6.**
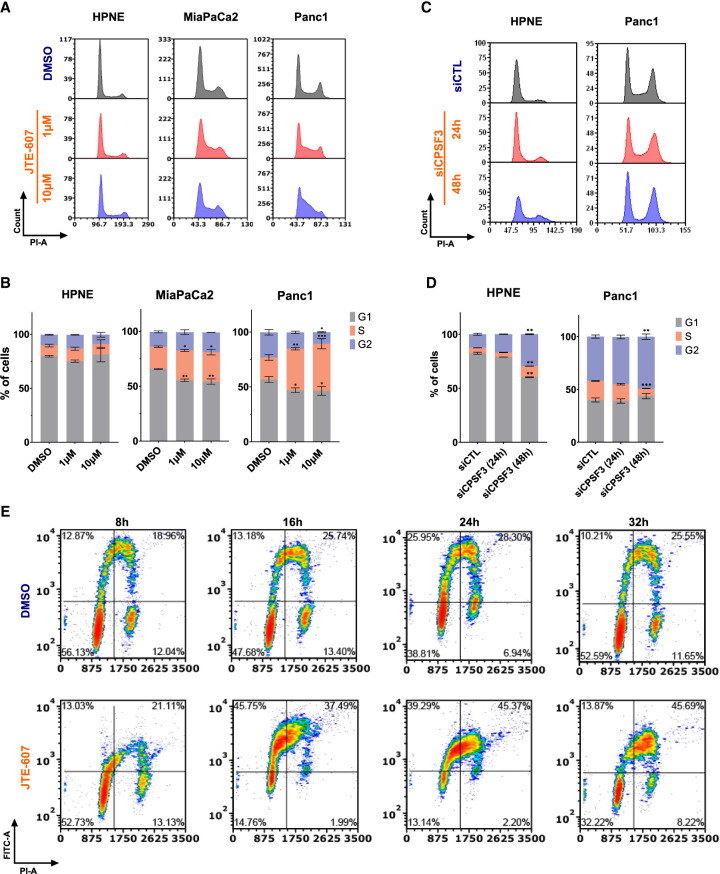
JTE-607 impairs cell cycle progression by inducing S-phase arrest. (*A*, *B*) Cell cycle distribution and quantification of HPNE, MiaPaCa2, and Panc1 cell lines treated with 1–10 µM JTE-607. (*) *P* < 0.05, (**) *P* < 0.001, (***) *P* < 0.0001, two-way ANOVA with Dunnett's multiple comparisons test. (*C*, *D*) Cell cycle distribution and quantification of HPNE and Panc1 cell lines upon transient *CPSF3* knockdown by siRNA after 24 h of transfection. (siCTL) Nontargeting control siRNA. (*) *P* < 0.01, (**) *P* < 0.001, two-way ANOVA with Dunnett's multiple comparisons test. Quantification in *B* and *D* is the number of cells in the S-phase. (*E*) BrdU incorporation assay showing cell cycle population upon JTE-607 treatment. The *lower left* quadrant represents the G1 population. The *lower right* quadrant represents the G2 population. The *top* two quadrants represent S-phase populations; early S-phase (*left*) and late S-phase (*right*).

Finally, we sought to determine how JTE-607 led to defects in cell viability. As RD histones are required for cell cycle progression, we assessed the effects of JTE-607 on cell cycle distribution. In immortalized control HPNE cells, JTE-607 had no impact on cell cycle distribution (Fig. 6A,B). In contrast, JTE-607 arrested Panc1 and MiaPaCa2 PDAC cells in the S-phase of the cell cycle within 24 h (Fig. 6A,B). To determine the impact of *CPSF3* knockdown on the cell cycle, we transiently knocked down CPSF3 with siRNA in HPNE and Panc1 cells (Supplemental Figs. S2D and S10G). *CPSF3* knockdown-induced cell cycle arrest in Panc1 cells with minimal effect on HPNE cells (Fig. 6C,D). However, unlike CPSF3 inhibition-induced cell cycle arrest at S-phase, *CPSF3* knockdown cells are arrested at G2 (Fig. 6D; Supplemental Fig. S12A,B). This pattern of cell cycle arrest is different from that induced by JTE-607 and does not resemble cell cycle arrest induced by histone defects in previous studies. This indicates that *CPSF3* knockdown-induced phenotype is indeed distinct from CPSF3 inhibition. To more specifically investigate the timing and extent of S-phase arrest upon JTE-607 treatment, we examined BrdU incorporation in a time-dependent manner (Fig. 6E). We found that JTE-607 arrests cells in the early to mid-S-phase of the cell cycle within 8 h. By 24 h, the majority of cells are arrested in S-phase. As arrest in S-phase in transformed cells can result in cell death, we assessed whether JTE-607 induces apoptosis in our PDAC cells by measuring caspase-3 and -7 activities. We found that JTE-607 did not significantly induce apoptosis at time points where cells are mainly arrested at S-phase as compared with the positive control Doxorubicin (Supplemental Fig. S13A–F). Overall, JTE-607 destabilizes chromatin and attenuates PDAC cell proliferation through S-phase cell cycle arrest.

## DISCUSSION

Our study has several clinical implications. First, we show that *CPSF3* expression is dysregulated in PDAC and high expression correlates with poor prognosis. This is consistent with similar findings across the cancer landscape, where *CPSF3* has been reported to be a predictor of unfavorable prognosis in lung and liver cancers (Ning et al. 2019; Li et al. 2021). While several studies have experimentally manipulated various mRNA processing factors and determined the phenotypic impacts, little is known about the function of *CPSF3* in disease, particularly cancer. This is noteworthy for several reasons. First, CPSF3 is the enzymatic component of the CPA and histone mRNA processing machineries, and is thus a potentially druggable target. Second, despite acting in the same complex, the knockdown of other CPA and histone mRNA processing factors can have opposing impacts on APA and histones as well as cellular phenotypes (Tan et al. 2017; Chen et al. 2018; Park et al. 2018; Pettinati et al. 2018; Zhang and Zhang 2018; Fang et al. 2020; Li et al. 2020). Recently, homozygosity in *CPSF3* missense variants was found to cause intellectual disability and embryonic lethality in humans. However, these phenotypes were completely absent in the heterozygous carriers (Arnadottir et al. 2022). In cancer cell line models, *CPSF3* is essential for cell proliferation when knocked out completely by CRISPR; however, *CPSF3* is not an essential gene upon shRNA-mediated partial knockdown (www.depmap.org). This suggests that pharmacological targeting of such an essential gene may be biologically feasible. In support of this hypothesis, we show that knockdown of *CPSF3* blocks PDAC cell proliferation and tumor growth. However, *CPSF3* knockdown does not affect cell proliferation of immortalized control cells suggesting its essentiality in highly proliferative cells. This is consistent with a recent report where sensitivity to CPSF3 inhibition is determined by high CPA activity and proliferation rate (Cui et al. 2023). Furthermore, we show that CPSF3 inhibition does not impair cell cycle progression or proliferation of immortalized control pancreatic epithelial cells, and the CPSF3 inhibitor JTE-607 is nontoxic in humans. Therefore, inhibition of CPSF3 may preferentially target transformed cells.

Recently, two groups independently demonstrated that CPSF3 is the target of the small molecule JTE-607 (Kakegawa et al. 2019; Ross et al. 2020). JTE-607 was first identified over 20 yr ago as a cytokine synthesis inhibitor; however, the direct molecular target remained elusive. Despite the lack of a defined mechanism, JTE-607 was tested in a Phase I dose-escalation trial in healthy human volunteers, with no serious adverse effects (Borozdenkova et al. 2011). Therefore, despite inhibiting an essential enzyme responsible for processing pre-mRNAs, JTE-607 is not uniformly toxic in humans. This property, coupled with our data demonstrating JTE-607's antiproliferative effects on cancer cells, supports the contention that targeting CPSF3 is a feasible prospect in PDAC. In humans, endotoxin-induced production of C-reactive protein, IL-10, and IL-1ra was inhibited by JTE-607 (Borozdenkova et al. 2011). In animal models, JTE-607 inhibited the production of proinflammatory cytokines, prevented endotoxin shock, and attenuated artificially induced lung and heart injury (Kakutani et al. 1999; Ryugo et al. 2004; Asaga et al. 2008). JTE-607 has also been used in models of AML and Ewing's sarcoma and showed growth inhibitory activity both in vitro and in vivo (xenograft models) (Uesato et al. 2006; Tajima et al. 2010; Ross et al. 2020). However, these studies were limited to leukemia and sarcoma models, with no efficacy shown for epithelial-derived tumors. Therefore, the potential for CPSF3 as a therapeutic target in adenocarcinoma was an open question. Now, we show that JTE-607 preferentially blocks the proliferation of PDAC cell lines, sparing immortalized control cell lines, including epithelial cells and fibroblasts. The mechanisms underlying this difference in sensitivity are currently unknown but may relate to variability in basal proliferation rate. We tested this hypothesis and showed that sensitivity to JTE-607 is associated with cells’ proliferative state. As JTE-607 is a pro-drug that requires intracellular activation by CES1, it is possible that differences in activation of the drug between different cell lines determine the strength of proliferative inhibition. However, JTE-607 sensitivity was found to be independent of CES1 expression levels (Ross et al. 2020). Finally, even though JTE-607 was first described as an inhibitor of cytokine synthesis, our RNA-seq analysis did not show an enrichment of such pathways. One possible explanation is that JTE-607 action is cell type dependent. The effects of JTE-607 in different cellular contexts and cell states warrant further investigation.

While several recent reports have linked *CPSF3* loss to defects in tumor cell growth, no study has mechanistically connected *CPSF3* to APA dysregulation. Genetic manipulation of CPA factors has been shown to alter APA patterns, dysregulate gene and protein expression, and drive cancer phenotypes (Masamha et al. 2014; Zhang et al. 2017; Brumbaugh et al. 2018; Chen et al. 2018; Park et al. 2018; Tan et al. 2018; Zhang and Zhang 2018; Chu et al. 2019; Xiong et al. 2019; Fang et al. 2020; Li et al. 2020, 2021). However, APA dynamics upon inhibition of CPSF3 activity has not been investigated. We now demonstrate that both *CPSF3* knockdown and inhibition result in APA in PDAC cells. Strikingly, CPSF3 influences APA in distinct patterns based on the mode of disruption. DAGs upon *CPSF3* knockdown and inhibition are different with only two genes commonly altered in both conditions. Additionally, we find that CPSF3 inhibition induces more lengthening events than *CPSF3* knockdown. While such observation has not been reported for CPSF3, this finding is consistent with a previous study where *CLP1*, another CPA factor, mediates distinct CPA patterns when lost versus when mutated (LaForce et al. 2022). The mechanistic differences underlying the *CPSF3* knockdown and inhibition effects raise several important questions. As CPSF3 is an integral subunit of the CPA complex, the effect of *CPSF3* knockdown and inhibition on the proper recruitment of other complex components was not previously known. We demonstrated that *CPSF3* knockdown, but not inhibition, may alter the stability of CPA complex components. Importantly, however, the discrepancies between *CPSF3* knockdown and inhibition extend to the expression of CPA factors at the protein, but not the mRNA level. *CPSF3* knockdown, but not inhibition, dysregulates the protein expression of CPA factors. The fact that basal protein levels of CPA factors are dysregulated may explain the divergence in APA patterns and gene expression alterations. This conclusion, however, is limited to the few probed CPA complex components, and further study is required for the remaining CPA complex subunits. Furthermore, whether *CPSF3* knockdown and inhibition distinctly influence PAS selection has not been previously studied. Here, we demonstrate that DAGs upon *CPSF3* knockdown and inhibition possess different motifs surrounding the PAS. Such differences have been shown to influence PAS selection thus inducing distinct APA patterns (Brown and Gilmartin 2003; Martin et al. 2012). Although *CPSF3* knockdown and inhibition affect APA differently, it remains difficult to delineate the molecular mechanism solely by computational means. It is possible that limitation of the motif algorithm may account for the differences in the enriched motifs.

JTE-607 attenuates cell proliferation in AML and Ewing's sarcoma through increasing R-loop formation and down-regulating the expression of DNA damage response genes (Ross et al. 2020). R-loops are DNA:RNA hybrids that form as a result of aberrant transcription, a characteristic of cancers with genetic rearrangements such as AML and Ewing's sarcoma (Gorthi et al. 2018; Luo et al. 2022). R-loops increase in models with mRNA CPA defects (Stirling et al. 2012), suggesting that the sensitivity of AML and Ewing's sarcoma to JTE-607 may be a consequence of high basal levels of R-loops, which eventually accumulate, leading to DNA damage and genomic instability. In our study, GSEA did not reveal changes in DNA damage response pathways upon *CPSF3* knockdown or inhibition in PDAC cells. Therefore, we propose that CPSF3 regulates cell proliferation through distinct mechanisms in AML and Ewing's sarcoma relative to PDAC. In PDAC cells, we find that JTE-607 impairs the processing of proliferation-dependent (RD) histone mRNAs. This is consistent with the role of CPSF3 in the HCC (Sullivan et al. 2009b; Yang et al. 2013, 2020; Sun et al. 2020; Gutierrez et al. 2021). Defects in the HCC have been shown to reduce the availability of RD histones (Zhao et al. 2004; Sullivan et al. 2009a,b; Armstrong and Spencer 2021). However, prior to now, no studies have described the effect of CPSF3 inhibition on HCC activity. Depletion of many HCC genes led to an accumulation of histone readthrough transcripts in the nucleus (Wagner et al. 2007; Romeo et al. 2014). Similarly, we find extensive transcript readthrough in RD histone mRNAs, but not RI histone mRNAs upon JTE-607 treatment in PDAC cells. In accordance with a previous study, *CPSF3* knockdown did not induce RD transcriptional readthrough (Pettinati et al. 2018). Importantly, neither *CPSF3* knockdown nor inhibition-induced histone transcriptional readthrough in immortalized control cells. This is consistent with the notion that slowly proliferating cells do not have high levels of RD histone transcription. In accordance with this model, we find that JTE-607, but not *CPSF3* knockdown, decreases mRNA levels of core histones in PDAC cells. The failure of *CPSF3* knockdown to inhibit histone gene expression may be due to the fact that a very small fraction of the total CPSF3 is present in the low abundance histone processing complex, and that complex may have a high affinity for the mCF subcomplex. On the other hand, even though it is possible that the reduction in core histone mRNA levels with JTE-607 can be attributed to defects in histone processing, a potential explanation for such reduction in histone mRNA is that the rate of cell growth has been reduced by JTE-607. Any mechanism that slows cell growth will also reduce the levels of histone mRNA. It is also possible that readthrough transcription was only identified for RD histones in PDAC cells because they are abundantly transcribed. Therefore, whether this reduction of core histone mRNA levels is a direct effect of the inhibition of CPSF3 on histone mRNA processing requires further study. Additionally, although inhibition of CPSF3 will result in the production of some unprocessed histone mRNA (i.e., readthrough), it might also result in some polyadenylated histone mRNAs, or misprocessed histone mRNA (Lyons et al. 2016). Furthermore, it is possible that there is a global transcriptional readthrough upon *CPSF3* knockdown and inhibition. Knockdown of *CPSF3* results in readthrough of most transcripts that are normally polyadenylated (Eaton et al. 2018, 2020). In addition, JTE-607 causes widespread transcriptional readthrough in HeLa and HepG2 cells (Cui et al. 2023). However, because these readthrough transcripts are very unstable, we were not able to detect them in our bulk RNA-seq data. Therefore, sequencing of nascent RNA is needed to assess the global impact on transcriptional readthrough.

Several studies have shown the effect of 3′-end mRNA processing on chromatin integrity. For example, JTE-607 increases accumulation in R-loops, DNA damage, and thus genomic instability (Ross et al. 2020). Additionally, inhibition of CPSF4 PAS recognition upon influenza infection by the NS1 protein causes RNA Polymerase II readthrough that leads to widespread changes in genome architecture dependent on NS1 (Heinz et al. 2018). We demonstrate that JTE-607 decreases core histone levels. Limited histone supplies destabilize chromatin through disruption of nucleosome assembly (Günesdogan et al. 2014). Chromatin is opened and destabilized since cells are in the S-phase replicating DNA and not producing enough histones to occupy it. We find that JTE-607 destabilizes chromatin in PDAC but not immortalized control cells, and derepresses heterochromatin-mediated gene expression silencing.

Expression of RD histones increases ∼30- to 50-fold during DNA synthesis (Marzluff and Pandey 1988; Osley 1991). The life cycle of these core histone genes starts late in G1 through the mid-S-phase of the cell cycle and degradation occurs at the late S-phase (Marzluff et al. 2008; Mendiratta et al. 2019). Silencing of the HCC core component *FLASH* induces S-phase arrest in HeLa cells (Barcaroli et al. 2006). We find that JTE-607 arrests cells in the S-phase of the cell cycle, with cells slowly cycling through the early mid-S-phase but failing to progress through the late S-phase. This is consistent with a previous study where depletion of the histone chaperone *ASF1* disrupts progression through mid to late S-phase (Groth et al. 2005). Importantly, silencing of *MBLAC1*, an endonuclease selective for 3′ processing of RD histone pre-mRNAs, significantly impairs cell cycle progression during S-phase (Pettinati et al. 2018). In addition, the knockdown of *CSTF2*, a gene with dual functions in CPA and histone pre-mRNA processing, delays progression through the S-phase, but its expression is highly dependent on the cell cycle stage (Romeo et al. 2014). The same study showed that *CPSF3* expression is not cell cycle regulated, suggesting that the histone phenotype we observe may be driven by CPSF3 inhibition and not merely a consequence of cell cycle arrest. However, it is possible that the effect of JTE-607 on histone mRNA levels is cell cycle regulated since the arrest in the S-phase results in rapid degradation of histone mRNA which would quickly lower histone mRNA levels. Although *CPSF3* knockdown induced cell cycle arrest, the pattern of cell cycle arrest is distinct from that induced by JTE-607 in our study and by histone disruption in previous reports. While our manuscript was under review, a publication reported that JTE-607 leads to DNA damage and S-phase crisis in HeLa and HepG2 cells (Cui et al. 2023). While JTE-607 induced S-phase arrest in PDAC cells, we did not see changes in DNA damage response pathways upon *CPSF3* knockdown or inhibition by GSEA. In fact, JTE-607 did not induce significant levels of apoptosis in our PDAC cells. Therefore, our findings suggest that JTE-607 mediates its growth-attenuating phenotype by arresting cells in the S-phase, possibly through reducing histone supplies thereby blocking cell cycle progression. In conclusion, our study has revealed the role of CPSF3 in pancreatic cancer and uncovered a new mechanism by which CPSF3 regulates cell proliferation.

There are several limitations to this study that warrant further investigation. First, there are clearly changes in the levels of some polyadenylated mRNAs which likely contribute to the cell proliferation deficiency, as well as some changes in APA which may contribute. Although around 1800 genes are altered in expression, only a small number show changes in APA. The contribution of CPSF3 inhibition to changes in PAS selection and the resultant effect on gene expression require further study. Although *CPSF3* knockdown and inhibition affect APA differently, it remains difficult to delineate the molecular mechanism solely by computational means. Additionally, a main limitation in comparing *CPSF3* knockdown and inhibition is that these approaches occur across different timescales. While we address this for RD histone readthrough, the different timescales may affect other observed differences in the levels of gene expression and APA. While our experiments detected transcriptional readthrough upon JTE-607 treatment, this does not necessarily mean that those transcripts are unprocessed RNAs. Rather, they fail to terminate RNA polymerase II but still they could be processed, a possibility that needs further experimental investigation. Also, it is possible that the limitation of the motif algorithm may account for the differences in the consensus signals. While our cell line models did not show APA alterations of PDAC-associated genes, we think this may be attributed to the heterogeneity of PDAC tumors, and analysis of APA using patient-derived single-cell RNA-seq data is underway to address this issue. Although *CPSF3* is an essential gene in all cells including immortalized control cells, it is likely that the relatively slow-growing cells upon *CPSF3* knockdown have adapted to grow with reduced levels of CPSF3. Furthermore, our analysis provides new insight into the mechanisms underlying JTE-607 target specificity. Next, it remains an open question how JTE-607 up-regulates the expression of a subset of genes. It is a possibility that JTE-607-induced relaxation of chromatin structure may result in aberrant transcription. Similarly, even though histone mRNA transcription factors are not altered at the level of APA or gene expression, open chromatin structure may facilitate transcription of suppressors of histone mRNA transcription, or interaction with suppressive elements. While these transcription factors bind to histone gene promoters, the fact that some of these are involved in the expression of many other genes must be taken into consideration. Also, it is important to keep in mind that histone gene transcription requires cyclin E/cdk2 (Zhao et al. 2000), which itself is a cell cycle regulator. Although the specificity of JTE-607 for CPSF3 has been supported by robust experimental validation in multiple studies, it is possible that off-target effects may occur. However, we note that the effects of JTE-607 on S-phase arrest and histone mRNA processing are similar to those produced upon depletion of the HCC component CSTF2 (Romeo et al. 2014).

## MATERIALS AND METHODS

Full details on all methods are available in the Supplemental Material.

### Cell lines and in vitro culture

HEK293T, MiaPaCa2, Panc1, Suit2, Human immortalized C7 CAFs, and PancPat CAFs cells were cultured in complete DMEM media. Nontransformed pancreatic cell line HPNE and HPDE cells were cultured in modified media. All cell lines were cultured at 37°C with 5% CO_2_ and tested negative for mycoplasma.

### Generation of CPSF3 knockdown cells

Cells were either stably knocked down using shRNA or transiently silenced using siRNA.

### RNA isolation and quantitative PCR

Cells were lysed with TRIzol reagent. RNA was then isolated and cDNA was synthesized. qPCR was conducted with SYBR Green PCR primers mixed with iTaq Universal SYBR Green Supermix and run on the CFX Connect System (Bio-Rad).

### Immunoblotting

Whole-cell lysates were lysed using RIPA lysis buffer with protease inhibitors, boiled at 95°C for 5 min, and resolved by SDS-PAGE. Proteins were transferred to nitrocellulose membranes, blocked with 5% nonfat dry milk in 1× TBST, and incubated with primary antibodies overnight at 4°C. Membranes were incubated with HRP-conjugated secondary antibodies at room temperature for 1 h, and Pierce ECL Western Blotting Substrate was used for chemiluminescent detection.

### Proliferation and clonogenicity assays

For proliferation experiments, cells were seeded into a white 96-well plate, and cell proliferation was measured on days 0, 2, 4, and 6. For clonogenicity assays, cells were seeded into a six-well plate, and colony area was measured after 11 d.

### Xenograft experiments

Animal experiments were approved by the Roswell Park Institutional Animal Care and Use Committee. MiaPaCa2 cells infected with shNTC and sh1 CPSF3 constructs were injected subcutaneously into the flanks of 8-wk-old NOD/SCID/IL2Rγ^−/−^ (NSG) mice. Tumor volume was determined by caliper measurements obtained in two dimensions and calculated as width^2^ × length/2 twice a week.

### Cell cycle analysis

Cells were trypsinized, fixed with 70% ethanol, washed with 1× PBS, and incubated with RNaseA at 37°C for 1 h. Propidium iodide was added and cells were analyzed by FACS at 488 nm.

### BrdU incorporation assay

Cells were cultured and incubated with BrdU for 4 h, rinsed, trypsinized, and permeabilized in 70% ethanol. Next, cells were pelleted, and DNA was hydrolyzed in 2N HCl and then neutralized with 0.1M sodium tetraborate. Cells were pelleted and incubated with Anti-BrdU-FITC. Cell pellets were then washed and resuspended in RNaseA and PI and incubated at room temperature for 30 min in the dark. Cells were then analyzed by flow cytometry.

### RNA-sequencing

For each condition, three biological samples were sequenced. Cell pellets were collected and sent to Roswell Park Genomic Shared Resources for RNA sequencing. Data were analyzed by the Roswell Park Bioinformatics Shared Resource.

### Bioinformatics analyses

Differential expression analyses were performed with DESeq2 (v1.36.0) (Love et al. 2014). For 3′-UTR, APA was analyzed using PolyAMiner-Bulk (Jonnakuti et al. 2023). For motif enrichment analysis, ungapped motifs of recurring fixed-length patterns in our sequence data sets were called using the STREME methodology (Bailey et al. 2015).

### Statistical analyses

Experimental findings were obtained from at least two independent experiments. *P* < 0.05 was considered statistically significant.

## DATA DEPOSITION

Sequencing data sets generated in this study have been deposited into the NCBI Gene Expression Omnibus with the accession number GSE252667 (GEO; https://www.ncbi.nlm.nih.gov/geo/).

## SUPPLEMENTAL MATERIAL

Supplemental material is available for this article.

## Figures and Tables

**Abdulrahman A. Alahmari:**
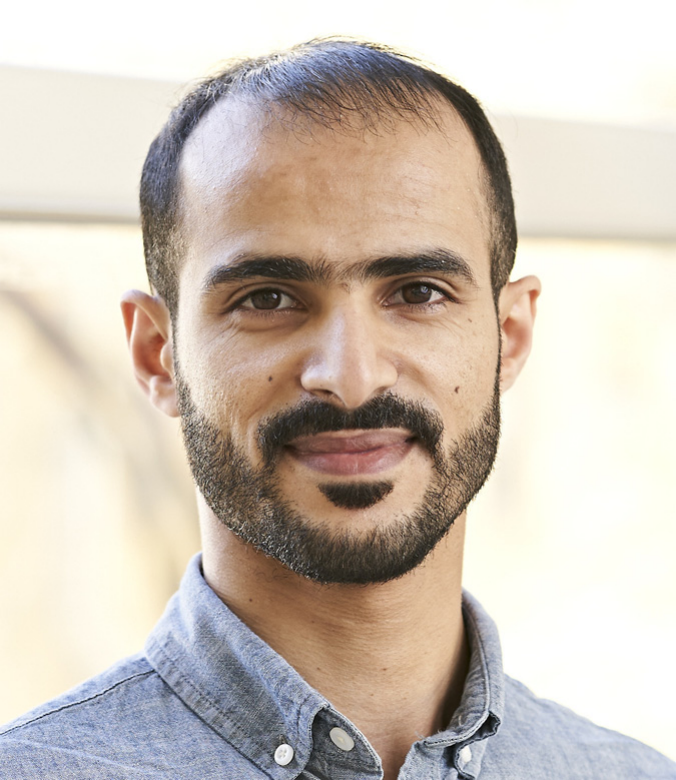


## MEET THE FIRST AUTHOR

***Meet the First Author(s)* is an editorial feature within *RNA*, in which the first author(s) of research-based papers in each issue have the opportunity to introduce themselves and their work to readers of *RNA* and the RNA research community. Abdulrahman A. Alahmari is the first author of this paper, “CPSF3 inhibition blocks pancreatic cancer cell proliferation through disruption of core histone mRNA processing.” Abdulrahman earned his B.S. in medical laboratory sciences at King Khalid University, Saudi Arabia in 2013 and an MSc in biomedical sciences at Quinnipiac University in 2016. Subsequently, he did postgraduate research in the laboratory of Dr. Fred Gorelick at Yale University. Currently, he is a senior PhD student in the Cancer Sciences PhD program, department of Cancer Genetics and Genomics, at Roswell Park Comprehensive Cancer Center, Buffalo, NY.**

**What are the major results described in your paper and how do they impact this branch of the field?**

This study shows the potential of targeting CPSF3 in pancreatic cancer. Specifically, chemical inhibition of CPSF3 preferentially attenuates cancer cell proliferation by disrupting proliferation-dependent histone mRNA processing, without affecting normal cells. This finding holds significant importance as many existing cancer therapies lack selectivity for cancer cells. Consequently, this research enhances the viability of targeting mRNA processing as a potential avenue for cancer treatment.

**What led you to study RNA or this aspect of RNA science?**

The regulation of gene expression is significantly influenced by RNA processing. Recently, there has been a growing recognition of dysregulated RNA processing in cancer. This underscores the significance of investigating changes in RNA processing in cancer to comprehend the underlying mechanisms and devise innovative therapeutic strategies. Despite the examination of various RNA processing genes in relation to cancer, a considerable number of those genes cannot be targeted for therapy. Consequently, we focused on CPSF3, an RNA processing factor that is therapeutically targetable, particularly in the context of pancreatic cancer.

**If you were able to give one piece of advice to your younger self, what would that be?**

I would advise my younger self that both patience and optimism are indispensable qualities for a scientist. I would also advise my younger self that the synergy of dedication and teamwork is interdependent. While dedication alone may propel you forward, its impact is limited without the collaborative strength of teamwork. Conversely, teamwork can streamline processes, but its efficacy diminishes without the fuel of individual dedication.

**Are there specific individuals or groups who have influenced your philosophy or approach to science?**

My PhD mentor, Dr. Michael Feigin, profoundly shaped my scientific approach. His work ethics and dedication to research served as a source of inspiration, fostering a deep commitment in my own scientific journey. Engaging in discussions and receiving constructive feedback from him further refined my critical thinking skills. Importantly, Dr. Feigin granted me the intellectual freedom I needed during my research, enabling not only the exploration of a myriad of novel ideas but also instilling a fervent passion for science within me.
